# Empowering sickle cell disease care: the rise of *TechnoRehabLab* in Sub-Saharan Africa for enhanced patient's perspectives

**DOI:** 10.3389/fresc.2024.1388855

**Published:** 2024-06-27

**Authors:** Paul Muteb Boma, Suzanne Kamin Kisula Ngoy, Jules Mulefu Panda, Bruno Bonnechère

**Affiliations:** ^1^ Reference Centre for Sickle Cell Disease of Lubumbashi, Institut de Recherche en Science de la Santé, Lubumbashi, Democratic Republic of the Congo; ^2^ Nursing Department, Higher Institute of Medical Technology, Lubumbashi, Democratic Republic of the Congo; ^3^ Department of Surgery, Faculty of Medicine, University of Lubumbashi, Lubumbashi, Democratic Republic of the Congo; ^4^ REVAL Rehabilitation Research Center, Faculty of Rehabilitation Sciences, University of Hasselt, Hasselt, Belgium; ^5^ Technology-Supported and Data-Driven Rehabilitation, Data Science Institute, University of Hasselt, Hasselt, Belgium; ^6^ Department of PXL—Healthcare, PXL University of Applied Sciences and Arts, Hasselt, Belgium

**Keywords:** sickle cell disorders, rehabilitation, technology-supported, rehabilomics, virtual reality, serious games

## Abstract

Sickle-cell Disease (SCD) is a major public health problem in Africa, and there are significant obstacles to its comprehensive management, particularly in terms of access to appropriate healthcare. This calls for inventive approaches to improve patients' prospects. Among the major challenges to be met are the primary and secondary prevention of certain serious complications associated with the disease, such as neurocognitive, motor and respiratory functional disorders. This perspective argues for the rapid creation of specific, cost-effective, technology-supported rehabilitation centres to advance SCD care, identify patients at high risk of stroke and implement tailored rehabilitation strategies. The *TechnoRehabLab* in Lubumbashi illustrates this shift in thinking by using cutting-edge technologies such as virtual reality (VR), serious games and mobile health to create a comprehensive and easily accessible rehabilitation framework. Diagnostic tools used to perform functional assessment can be used to identify cognitive, balance and walking deficits respectively. Transcranial Doppler enables early detection of sickle cell cerebral vasculopathy, making it possible to provide early and appropriate treatment. VR technology and serious games enable effective rehabilitation and cognitive stimulation, which is particularly advantageous for remote or community-based rehabilitation. In the context of African countries where there is a glaring disparity in access to digital resources, the *TechnoRehabLab* serves as a tangible example, demonstrating the flexibility and accessibility of technology-assisted rehabilitation. This perspective is an urgent call to governments, non-governmental organisations and the international community to allocate resources to the replication and expansion of similar facilities across Africa.

## 1 Introduction

Sickle cell disease (SCD) is associated with severe morbidity, particularly due to acute and chronic complications affecting several organs (1). Some of these complications are disabling, such as stroke and osteoarticular damage (2, 3), and increase the social and financial burden of the disease (4). It is estimated that 8.3% of patients with SCD will suffer a stroke at a young age (5). Ultimately, these patients suffer motor and cognitive sequelae leading to severe disability. SCD is also associated with aseptic necrosis of the femoral heads, leading to complications of the musculoskeletal system that may ultimately necessitate the fitting of prostheses (6). Improving this morbidity depends above all on preventive measures, the primum movens of which is early detection of the disease, which ensures appropriate treatment before the first complications arise. Prevention of infection and measures to promote a healthy lifestyle have improved the life expectancy of patients. These measures have been complemented by therapeutic advances, in particular the use of hydroxyurea, chronic transfusions with the possibility of oral iron chelation, and the use of erythrocytapheresis, bone marrow transplants and, more recently, gene therapy, although this is not yet available in current practice. Despite disparities in access to care, which tend to disadvantage certain social groups, particularly in the US (4), These therapeutic advances are widely available in developed and some emerging countries, but are not yet well implemented in Sub-Saharan Africa, where the majority of people with SCD live (7, 8). This is due to non-existent or unsuitable infrastructures, the fact that funding for health services is essentially dependent on direct out-of-pocket payments and external partners who have other priorities than SCD, and the inadequacy or non-existence of diagnostic services (9). However, over the last two decades there have been several initiatives that have led to an improvement in the provision of care for sickle cell patients. In Nigeria and Tanzania, haematopoietic stem cell transplantation units are operational to offer patients the possibility of curative treatment at an affordable cost to stem the burden of the disease, the cumulative cost of non-curative treatments of which is enormous in this context of scarce resources (10–12) and Ethiopia plans to have it by 2029 (13). These ambitious initiatives are certainly commendable, but they would not immediately apply to the largest number of sickle cell patients in Sub-Saharan Africa. The challenges remain enormous. And even in developed and emerging countries, not all sickle cell patients benefit from bone marrow transplantation. The most significant advances are to be found in the emergence of specialised centres or services in certain major cities in different countries. From Bamako to Dar-es-Salam, via Dakar, Brazzaville, Bangui, etc., the most significant advances are found in the emergence of specialised centres or services. These centres, together with the introduction of various university diplomas aimed at improving the knowledge and skills of healthcare professionals in the field of SCD, are vectors for improving the survival and quality of life of people with these patients (14). In the Democratic Republic of Congo, the first structure dedicated to SCD was created in 1974 within the National Research and Development Office. This structure was subsequently placed under the administrative supervision of the Institut de Recherche en Sciences de la Santé, whose mission is to provide services and training to find solutions to priority health problems, in particular SCD, malnutrition, malaria, and HIV/AIDS. It organizes the care of sickle cell patients at the Centre de Médecine Mixte et d'Anémie SS in Kinshasa and at the Centre de Référence de la Drépanocytose de Lubumbashi in its provincial branch. However, the diagnostic and therapeutic challenges to making a real impact on the course of SCD disease in Sub-Saharan Africa remain enormous.

Among the many challenges facing the Lubumbashi Sickle Cell Reference Center was identifying patients at risk of stroke and those with cognitive impairment. Additionally, there was a need to promptly establish rehabilitation services for individuals facing motor deficits post-stroke or aseptic necrosis of the femoral head, particularly after undergoing surgical drilling treatment. To address these challenges and enhance patient-centered care, the Institute forged a partnership with REVAL of Hasselt University, leading to the establishment of the *TechnoRehabLab* in Lubumbashi. This program outlines a north-south initiative aimed at enhancing services provided to SCD patients by implementing best practices that enhance care quality, mitigate treatment expenses, and ultimately improve patient well-being.

## 2 SCD rehabilitation dynamics in Africa

The complexities associated with tackling SCD in the African environment manifest as an intricate and varied task. The burden of SCD is worsened throughout the continent due to a combination of variables, such as inadequate availability of rehabilitative services, scarcity of resources, and the lack of specific therapies. But there is also a lack of guidelines and recommendations about the need of rehabilitation services for patients with SCD in the official recommendation of the Lancet Haematology Commission (15). Rehabilitation, physiotherapy, or physical medicine were hardly mentioned in this report. This omission is of particular concern, especially when considering the recent focus of WHO on global rehabilitation services and the prioritized management of disabilities within the health-care domain. This effort is highlighted in the establishment of the World Rehabilitation Alliance, aimed at addressing the escalating global need for comprehensive rehabilitation solutions (16). Unfortunately, this crucial component is notably absent from the Commission's recommendations despite its significance for patients (17).

The urgent necessity for efficient rehabilitation interventions is further emphasized by the increased occurrence of stroke among patients dealing with SCD, intensifying the need for a reevaluated and all-encompassing healthcare approach (18).

The lack of access to rehabilitation treatments in numerous African countries is evident (16), resulting in a significant gap between the requirements of individuals with SCD and the existing healthcare infrastructure (19). The limited availability of resources exacerbates the difficulties, leading to a healthcare environment that is not well prepared to meet the complex requirements presented by SCD (20). Without specific interventions, individuals with SCD are left to navigate a complicated healthcare system that often lacks the necessary specialist support to meet their distinct needs (21).

The high frequency of stroke among individuals with SCD creates an emergency situation, requiring the rapid deployment of appropriate interventions, including early rehabilitation (17, 18). Conventional methods of rehabilitation struggle with obstacles caused by a significant scarcity of healthcare specialists who specialize in SCD care. Furthermore, deficiencies in healthcare infrastructure, worsened by financial limitations, additionally impede the provision of prompt and specific rehabilitation services (22). The current paradigms need a thorough reassessment to tackle these systemic barriers and establish a more comprehensive, flexible, and patient-centered care approach (23).

The necessity for a comprehensive approach is clear, one that not only tackles the urgent medical requirements but also recognizes the socioeconomic issues that underlie the difficulties connected with SCD in Africa (24). In order to improve the treatment of these patients in Africa's healthcare system, it is important to carefully analyze the current obstacles and develop novel solutions. This will help create a more fair and effective approach to SCD management within the complex and highly underfinanced African healthcare environment.

## 3 The *TechnoRehabLab*

**Figure 1:**
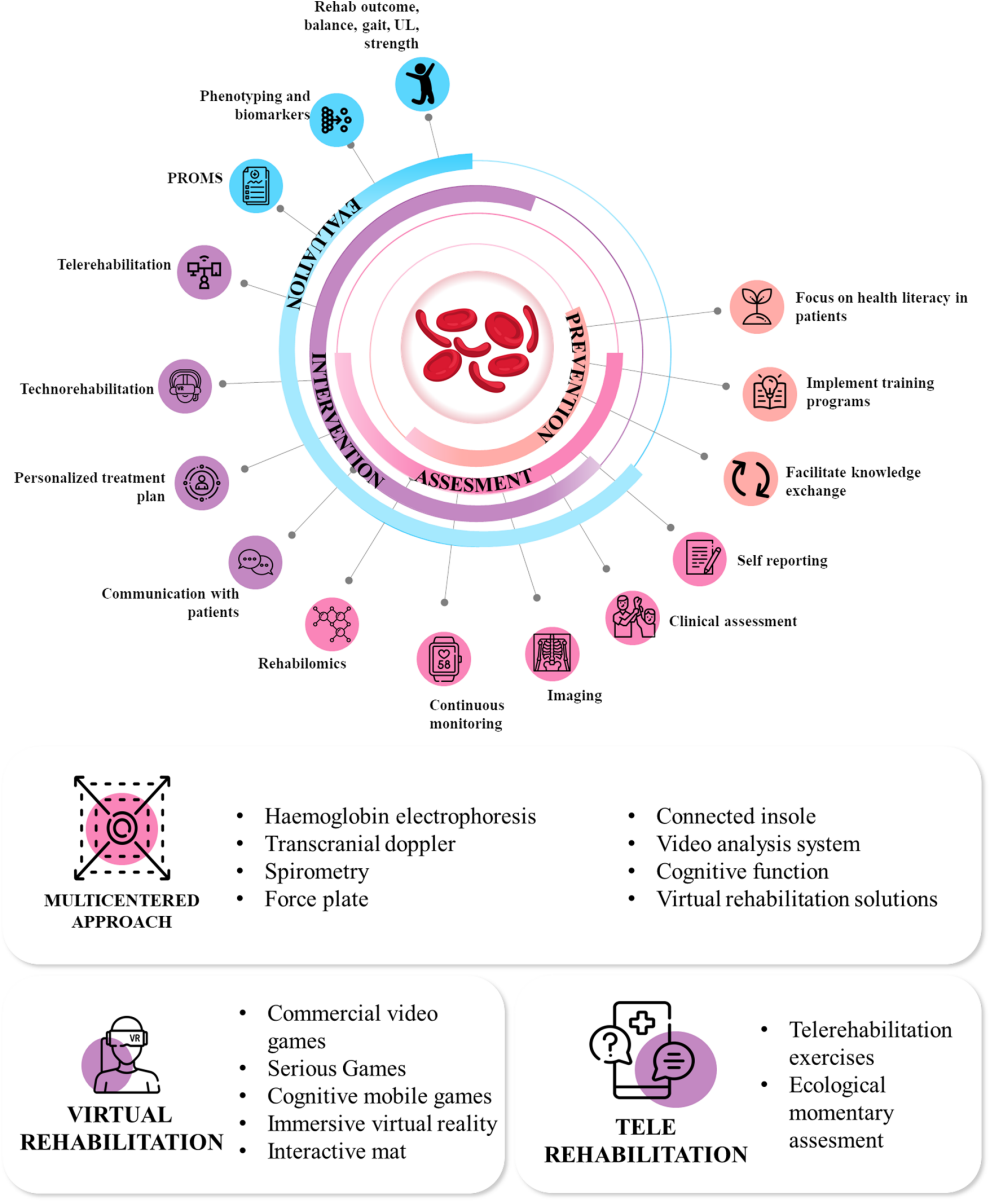
The different objectives, level of intervention and technologies available at the center.

The *TechnoRehabLab* in Lubumbashi is at the forefront of scientific innovation and practical application, addressing the significant issues associated with managing SCD in the African environment. This advanced facility, opened in November 2023, goes beyond traditional limits, combining technology and rehabilitation approaches to create a comprehensive framework for managing both cognitive and motor impairments of SCD. The *TechnoRehabLab* combines modern tools such as virtual reality (VR) and mobile technology to provide cost-effective and accessible rehabilitation services to SCD patients. Being highly patients-centred (25) and goal-oriented (26), these services are specifically designed to meet the individual needs of each patient. The technologies and services can be summarized into three key main components, complete list and descriptions of the technologies and service available are presented in Table 1 and summarized in Figure 1.

**Table 1:** Description of the technology available in the center.

Category	Technology	Description	Aim
Virtual rehabilitation	Commercial video games	Commercial video games such as Nintendo Wii, alone or in combination with the Balance Board, the Microsoft Xbox Kinect and the Nintendo Switch has been successfully tested and integrated in the rehabilitation program to increase both motor and cognitive functions (27).	• Improve balance and coordination • Restoring mobility • Decrease fatigue • Improve cognitive ability • Pain reduction
	Serious games	Serious games are video games that have been specifically developed for rehabilitation purposes. They are adapted to the specificity of the rehabilitation process and can be easily configured to each and every patients (28).	• Improve balance and coordination • Restoring mobility • Decrease fatigue • Improve cognitive ability • Pain reduction
	Cognitive mobile games	Brain training exercises have been increasingly assimilated into computerized training paradigms, spanning across various platforms including personal computers, gaming consoles, and, more recently, smartphones and tablets (29). This evolution in delivery mechanisms has widened the accessibility and utilization of cognitive training interventions, facilitating their integration into daily routines and enabling a broader demographic to engage in structured cognitive enhancement programs.	• Improve cognitive ability • Decrease fatigue
	Immersive virtual reality	Virtual Reality (VR) is characterized as a sophisticated human-computer interface, facilitating users’ exploration, interaction, and immersion in environments simulating real-world objects and events. This immersive experience is enriched by real-time feedback and augmented sensory inputs, providing users with comprehensive insights into their performance. VR technology encompasses a diverse array of hardware configurations, including computers, mobile device screens, and head-mounted displays, which collectively enable patients to engage in repetitive, intensive, and goal-oriented practice across various levels of immersion within virtual environments (30).	• Improve balance and coordination • Restoring mobility • Decrease fatigue • Improve cognitive ability • Pain reduction • Improve proprioception
	Interactive mat	The interactive mat is a pressure-sensitive rehabilitation mat equipped with sensors. It offers a variety of exercises aimed at improving balance, coordination, muscular strength, and mobility. All exercises can be tailored to the individual needs of patients (31).	• Improve balance and coordination • Restoring mobility • Decrease fatigue • Improve proprioception
Telerehabilitation	Telerehabilitation exercises	Telerehabilitation presents a novel approach to delivering rehabilitation services, offering patients the opportunity to engage in exercises more conveniently from the comfort of their homes. Leveraging information and communication technologies, telerehabilitation fosters seamless communication between healthcare professionals and patients, even when they are in remote locations. This innovative approach not only enhances accessibility to rehabilitation services but also empowers patients to perform exercises with greater ease and consistency, promoting improved outcomes and overall well-being (32).	• Increase adherence to home-based exercises ◯ Improve balance and coordination ◯ Restoring mobility ◯ Decrease fatigue ◯ Improve cognitive ability ◯ Pain reduction
	Ecological momentary assessment	SEMA (Smartphone Ecological Momentary Assessment) emerges as a solution tailored for the intensive longitudinal evaluation of pain, fatigue, motivation, and other pertinent factors in SCD patients (33). This method allows researchers and clinicians to gain real-time insights into the fluctuating experiences and challenges faced by children with SCD, facilitating tailored interventions and improving overall care strategies.	• Regular and ecological subjective measurement of quality-of-life related factor ◯ Fatigue ◯ Pain ◯ Motivation
Multidimensional assessment unit	Transcranial doppler	Transcranial Doppler (TCD) is a rapid and non-invasive diagnostic technique that can provide real-time measurements of the relative changes in cerebral blood velocity (CBV). Therefore, TCD is a useful tool in the diagnosis and treatment of clinical cerebrovascular diseases (CVDs). Specifically, TCD could be applied to evaluate occlusive CVD, assess collateral circulation in patients with ischemic stroke, and monitor cerebral vascular occlusion before and after thrombolysis as well as cerebral vasospasm (VSP) and microembolization signals after aneurysmal subarachnoid hemorrhage (SAH). Moreover, TCD could predict short-term stroke and transient cerebral ischemia in patients with anterior circulation occlusion treated with endovascular therapy and in patients with anterior circulation vascular occlusion (34).	• Utilization of a haemoglobin electrophoresis reader allows for fast and reliable identification • Swift quantification of various haemoglobin types including Hb A (normal), Hb S (sickle cell), Hb F (fetal), and Hb A2/C/E • Enables clinicians to discern relative concentrations of haemoglobin variants promptly • Facilitates comprehensive assessments crucial for precise diagnosis and tailored treatment strategies, particularly in hemoglobinopathies such as sickle cell disease
	Haemoglobin electrophoresis	The utilization of a haemoglobin electrophoresis reader presents an invaluable asset in the swift and accurate identification as well as quantification of various haemoglobin types, including but not limited to, Hb A (normal), Hb S (sickle cell), Hb F (fetal), and Hb A2/C/E. This advanced diagnostic tool enables clinicians to promptly discern and measure the relative concentrations of these haemoglobin variants, facilitating comprehensive assessments crucial for precise diagnosis and tailored treatment strategies, particularly in the context of hemoglobinopathies such as sickle cell disease (35).	• Rapid identification of diverse haemoglobin disorders • Confirmation of sickle cell syndromes diagnosis • Facilitates prompt initiation of appropriate management strategies • Enables timely interventions to mitigate associated complications and improve patient outcomes
	Spirometry	Spirometry is classically used to diagnose airway diseases in patients with SCD. This is crucial tool given that compromised pulmonary function stands as a primary contributor to the morbidity and mortality rates observed in pediatric SCD cases. By utilizing spirometry, clinicians can effectively evaluate respiratory function, aiding in the early detection and management of respiratory complications (36).	• Utilize spirometry as a classical diagnostic tool for identifying airway diseases in SCD patients. • Early detection and management of respiratory complications. • Evaluation and follow-up of the patients
	Force plate	Evaluation of both static and dynamic balance under different conditions (i.e., eyes open, closed, stable or unstable surfaces) (37), to monitor the evolution of the patients and offers personalized-rehabilitation exercises. The force plate also has a rehabilitation component offering serious games to improve biofeedback.	• Balance assessment • Evaluation of the risk of osteonecrosis • Proprioception deficit
	Connected insole	Gait analysis is a powerful technique for capturing human locomotion and accurately measure numerous related factors. The ability of this equipment to offer comprehensive insights on gait has made it an essential instrument in both clinical and scientific environments (38).	• Quantified gait analysis • Evaluation of the risk of osteonecrosis • Proprioception deficit
	Video analysis system	The development and evolution of video based analysis (marker less system) supported by artificial intelligence allow to perform simple yet validated functional assessment of the patients (39).	• Gross motor function assessment • Evaluation and follow-up of the patients
	Cognitive function	Fully automated and quantitative evaluation of various cognitive functions such as inspection time, reaction time, impulse control, short term memory, and executive function using tablets (40). The added value of this system is that the evaluations are not impacted by educational level of culture.	• Quantitative evaluation of cognitive function • Evolution through the rehabilitation process or evaluating the natural history of the disease • Stroke risk assessment
	Virtual rehabilitation solutions	One of the salient aspects of the new technologies used to support the rehabilitation process is that a lot of data can be collected to monitor the evolution of the patients and to provide them direct feedback. Serious games can be used to assess both motor (41, 42) and cognitive (43) functions.	• Continuous and more longitudinal assessment of the patients during the whole rehabilitation process.

### 3.1 Virtual rehabilitation

The use of new and affordable technologies goes beyond the scope of simple functional rehabilitation; it also introduces a paradigm shift by including an element of cognitive stimulation (44). Current modalities of rehabilitation include both supervised and unsupervised exercises, but advances in technology are opening new horizons in this field such as Virtual Reality (VR), Augmented Reality (AR), gamification, and telerehabilitation (45). This forward-thinking strategy encourages the highest possible level of patient engagement inside the comforting environment of their own homes. The selection of various technologies has been based on our expertise in technology-supported rehabilitation, as well as on different criteria including financial considerations, language support, offline usability, and geographical accessibility for telerehabilitation applications (46).

The *TechnoRehabLab* accommodates a wide range of rehabilitation requirements through the utilization of a variety of applications. These technologies improve patient compliance and successfully solve issues that are inherent to conventional rehabilitation paradigms (47).

### 3.2 Telerehabilitation services

Recognizing the existing digital divide, the *TechnoRehabLab* makes strategic advantage of the widespread use of mobile devices that are within a reasonable price range in Lubumbashi. Mobile health (mHealth) refers to the integration of mobile devices, including mobile phones and patients monitoring devices, into medical and public health practice. This approaches leverages the fundamental capabilities of mobile phones, encompassing short messaging system, as well as advanced technologies (3G, 4G and 5G), global positioning system and Bluetooth (48).

The seamless implementation of at-home training programs is made possible by these apps, which serve as an accessible way of providing telerehabilitation, guarantying the continuity of the care (49). This is especially beneficial for families that do not have access to long-term rehabilitation clinics.

### 3.3 Multidimensional assessment unit

For the purpose of facilitating the early detection of cerebral vasculopathy (50), the *TechnoRehabLab* makes use of cutting-edge diagnostic technologies such as transcranial doppler (TCD) (51). In the process of identifying patients who are at an increased risk of having a stroke, this evaluation plays a crucial role in prevention (52).

These diagnostic findings serve therefore as the basis for implementation of early intervention techniques, which may include continuous transfusion programs and therapy with hydroxyurea (53). It is important to note that these measurements are precisely adjusted to the personalized needs of patients, which represents a substantial change from the standard treatments that are designed to be universally applicable and highlights the significance of precision medicine in the management of SCD given the scarcity of resources. Not only does the incorporation of technology at each and every stage guarantee prompt interventions (54), but it also guarantees a more nuanced and individualized treatment trajectory for persons who are attempting to deal with the many issues that are posed by SCD (55).

## 4 Challenges and opportunities

The *TechnoRehabLab*, at the intersection of technology and rehabilitation, grapples with unique challenges and promising opportunities to increase both the quality and quantity of rehabilitation services in regions with limited human and financial resources.

### 4.1 Challenges

The limited accessibility of rehabilitation therapies presents a major obstacle in the management of SCD in Africa (56). This inadequacy worsens the already significant burden of SCD on individuals affected by it (57). The facility established in Lubumbashi has been assigned the critical mission of addressing and resolving this pervasive problem. The organization's mandate extends beyond conventional rehabilitation approaches, requiring innovative measures to effectively tackle the accessibility obstacles encountered by those coping with SCD. Important efforts also need to be done in order to monitor the efficacy of such intervention and to define local evidences supporting this. Therefore in parallel with the clinical training of the personal we are also forming them to research principles in order to collect high quality data and determine which interventions are the most suited for these patients in this particular context.

The center faces a substantial obstacle as a result of the scarcity of financial and infrastructural resources (19). The insufficient resources provide a substantial barrier to the efficient operation and potential growth of the *TechnoRehabLab* and the development of other centers. The Lab's mission is to address these resource limits, which is an important objective. Efficient strategic planning and innovative solutions are necessary to acquire the necessary resources, guaranteeing the uninterrupted provision of high-quality rehabilitation services. The *TechnoRehabLab* was established as a commissioned entity within a North-South collaboration. The funds were used towards acquiring necessary equipment and covering operational expenses, so guaranteeing patients unrestricted access to assessment and rehabilitation services without charge. Securing adequate funding is a significant obstacle in ensuring the long-term sustainability of this specialized center and preserving access to its services (58). There are requirements that must be fulfilled in terms of equipment to enhance the technological infrastructure for conventional rehabilitation, as well as in terms of accessibility to healthcare. The last point is particularly significant, as user fees serve as the primary means of funding healthcare in the Democratic Republic of Congo. This hinders the ability of underprivileged patients to receive high-quality healthcare since there is an imbalance in the allocation of resources, resulting in unequal access to care for everyone (59). The primary problem lies in acquiring the necessary resources to fully fund the operational expenses of the center. Currently the center is integrated whithin the Institut de Recherche en Sciences de la Santé, which also manages the *Centre de Référence de la Drépanocytose de Lubumbashi*. However, it is located in premises that have been generously provided by a partnering hospital.

In order to ensure the long-term viability of its operations, the organization needs a dedicated physical facility that is both feasible and well-suited to fully maximize the services it can provide to patients. This area should provide the necessary conditions for the installation of all essential utilities and guarantee uninterrupted access to electricity and the internet for the daily functioning of the facility. The lab also requires a diverse array of medical supplies and equipment in order to enhance its technical infrastructure and uphold the standard of care and effectiveness offered to patients (see Table 1). Having motivated and trained personnel is crucial for the functioning of the system. This necessitates continuous investment in the remuneration and training of healthcare professional (60). To ensure the long-term sustainability of this project, contact and regular discussions with the local Ministry of Health are underway to integrate these services into the universal health coverage for these patients.

### 4.2 Opportunities

In the context of managing SCD, the newly developed center plays a crucial role in providing a unique chance to create a comprehensive framework that surpasses conventional methods. By using technology, the lab establishes itself as an essential link in the chain of providing appropriate care for a complete solution that effectively addresses the complex problems associated with SCD.

Another really important aspect is the development of local research expertise in the center. It is indeed of the utmost importance to develop local evidences supporting local interventions. The disparity between the quantity of scientific and clinical researchers in low- and middle-income countries (LMICs) and their significant burden of diseases like sickle cell disease is compounded by the emigration of a substantial portion—up to 70%—of scientists seeking education and employment opportunities abroad (61). However, it's notable that while new technology-supported interventions have been validated primarily in high-income countries (HICs), it remains crucial for clinicians and researchers in LMICs to conduct studies to ascertain the applicability of these findings to their contexts. It is imperative that studies are conducted to assess the viability and effectiveness of these new interventions in LMIC settings. Only through such research can the clinical utility of these interventions be established, thereby paving the way for their reimbursement and widespread implementation (62).

The success of this center makes it a viable model that can be replicated in many places throughout Africa. The lab's exemplary status has the potential to influence not only Lubumbashi but also other regions throughout the continent by encouraging the creation of similar services aimed at resolving the difficulties associated with the management of neurocognitive and motor complications associated with SCD, but these facilities can also be used to provide (neuro)rehabilitation services to other highly prevalent pathologies such as for example stroke (63) and cerebral palsy (64). These can be services operating independently or integrated into the operation of centers dedicated to the management of SCD.

Furthermore, the use of technology in the *TechnoRehabLab* creates opportunities for groundbreaking rehabilitation methods. By employing, amongst other, VR and mHealth, the lab can provide solutions that are both innovative and patient-centric while still being cost-effective. This strategy is specifically designed to address the distinct situations and difficulties encountered by those coping with SCD. Although the lab faces substantial challenges presented here above, strategic initiatives, collaborative endeavors, and resolute dedication to patient-centered treatment have the potential to convert these obstacles into favorable circumstances. The pressing need for action involves harnessing the potential of technology-supported rehabilitation to transform patients' management in Africa.

This project is an important symbol of constructive progress, mandating the establishment of an effective health financing system that ensures individuals with SCD can access the appropriate care they need without being burdened by excessive costs. This initiative encourages governments, non-governmental organizations (NGOs), and the international community to allocate resources and actively support programs that can have significant and far-reaching impacts. In our case, a consortium of public and private partners is in action. It relies on the institutional and technical support of the Provincial Ministry of Health, which is involved in providing premises for the definitive establishment of the center and in supporting advocacy for the search for funding. The scientific and administrative managers of the Health Research Institute do their utmost to ensure that the project benefits from all the administrative facilities necessary for its optimal functioning and the improvement of the remuneration conditions of the staff assigned to this service. The entire civil society in the fight against SCD in the region is mobilized to support the center in its acceptance in the community and in its efforts to mobilize local and international resources.

## 5 Conclusion

The *TechnoRehabLab* in Lubumbashi serves as a clear example of how technology-based rehabilitation can effectively transform the way SCD is managed in limited-resourced environment. Immediate effort is necessary to duplicate and expand such projects throughout the African continent, initiating a significant change in healthcare delivery. It is crucial for governments, NGOs, and the international community to make significant investments in the establishment of economically feasible rehabilitation institutions that utilize technology. This investment is designed to guarantee fair and equal access to high-quality care for those dealing with SCD. At this time, it is necessary to take decisive action as we work towards a future where technology enables effective management of SCD, reduces the impact of stroke, and improves the quality of life for many people throughout the continent.

## Data Availability

The original contributions presented in the study are included in the article/Supplementary Material, further inquiries can be directed to the corresponding author.
